# Early Changes in Transcriptomic Profiles in Synaptodendrosomes Reveal Aberrant Synaptic Functions in Alzheimer’s Disease

**DOI:** 10.3390/ijms23168888

**Published:** 2022-08-10

**Authors:** Xueqi Qu, Li Lin, Wanying Yi, Changyu Sun, Yuewen Chen, Yu Chen

**Affiliations:** 1 Chinese Academy of Sciences Key Laboratory of Brain Connectome and Manipulation, Shenzhen Key Laboratory of Translational Research for Brain Diseases, The Brain Cognition and Brain Disease Institute, Shenzhen Institute of Advanced Technology, Chinese Academy of Sciences, Shenzhen–Hong Kong Institute of Brain Science–Shenzhen Fundamental Research Institutions, Shenzhen 518055, China; 2 Guangdong Provincial Key Laboratory of Brain Science, Disease and Drug Development, HKUST Shenzhen Research Institute, Shenzhen 518057, China; 3 University of Chinese Academy of Sciences, Beijing 100049, China

**Keywords:** neurodegenerative disease, local translation, synapse loss, synaptic transmission

## Abstract

Alzheimer’s disease (AD) is one of the most prevalent neurodegenerative disorders characterized by the progressive decline of cognitive functions, and is closely associated with the dysfunction of synapses, which comprise the basic structure that mediates the communication between neurons. Although the protein architecture and machinery for protein translation at synapses are extensively studied, the impact that local changes in the mRNA reservoir have on AD progression is largely unknown. Here, we investigated the changes in transcriptomic profiles in the synaptodendrosomes purified from the cortices of AD mice at ages 3 and 6 months, a stage when early signatures of synaptic dysfunction are revealed. The transcriptomic profiles of synaptodendrosomes showed a greater number of localized differentially expressed genes (DEGs) in 6-month-old AD mice compared with mice 3 months of age. Gene Ontology (GO) analysis showed that these DEGs are majorly enriched in mitochondrial biogenesis and metabolic activity. More specifically, we further identified three representative DEGs in mitochondrial and metabolic pathways—*Prnp*, *Cst3*, and *Cox6c*—that regulate the dendritic spine density and morphology in neurons. Taken together, this study provides insights into the transcriptomic changes in synaptodendrosomes during AD progression, which may facilitate the development of intervention strategies targeting local translation to ameliorate the pathological progression of AD.

## 1. Introduction

Alzheimer’s disease (AD) is the most common neurodegenerative disorder leading to dementia in the elderly. It is characterized by the deposition of intercellular insoluble amyloid-beta (Aβ) plaques [1,2,3], and intracellular neurofibrillary tangles (NFTs) comprising hyperphosphorylated tau (p-tau), in the brain [1,4]. Synapses are the functional unit of the nervous system that transduce information among neurons, and synapse loss is one of the early pathological changes contributing to cognitive decline in AD [5]. Moreover, this cognitive decline is strongly correlated with synapse degeneration in AD, including spine structure alteration, spine density reduction, and pre- as well as postsynaptic component changes at synapses [2,6,7,8]. Importantly, synaptic dysfunction in neurodegenerative disease is closely correlated with tenuous amnesic problems in early stage AD, which may represent a temporal window for intervening therapeutically before any irreversible damage occurs in the brain parenchyma [9]. The above findings are supported by ultrastructural and microscopic examination that have assessed hundreds of human synapses. However, synaptic molecular characterization is limited for AD.

mRNA localization, transport, and local translation at synapses play critical roles in synaptic plasticity [10]. The trafficking of mRNAs and localized synthesis of proteins in dendrites are important for neuronal activity [11]. In differentiated neurons, numerous mRNAs are dendritically enriched and proteins are locally synthesized, which enable rapid and robust synapse-specific responses to neuronal stimulation [12,13,14]. An investigation of fragile X syndrome revealed that the loss of local mRNA regulation in dendritic spines alters synaptic function and local protein synthesis-dependent plasticity [15]. Meanwhile, the study identified another local mRNA population in which the locally translated proteins are involved in local protein synthesis. Similarly, the abundance of synaptic GluA1—the key factor for mediating synaptic plasticity, through both the trafficking of GluA1 mRNA and local GluA1 synthesis, in neuronal dendritic spines—mediates rapid excitatory synaptic transmission [16]. Notably, synaptic failure is a pathological hallmark of AD, but the molecular characterization of synaptically localized mRNAs in AD remains largely unknown [9]. Therefore, it is important to identify the members and functions of synaptically localized mRNAs to gain further insights into the synaptic modulation of AD. 

Synaptodendrosomes, potential sources of dendritic RNAs, are subcellular fractions enriched in pinched-off, resealed dendritic spine structures [17]. They contain synaptosomes, synapse junctions, and postsynaptic components, which are attached to resealed dendritic fragments and dendritic spines to retain metabolic and enzymatic activities [17]. RNA expression profiles in synaptosomes show that a cluster of mRNAs are specifically enriched in the synaptic fractions of adult mice [18]. Analysis of these synaptically localized transcripts reveal diverse modes of regulation of synaptic function in the pathogenesis of AD [18]. Among these example genes, a deeper investigation reported that the differentially localized and expressed mRNA levels of Grin2c could potentially link the regulation of synaptic strength distribution to the expression of synaptic plasticity [19]. 

The above evidence suggests that mRNAs localized in neuronal dendrites are involved in the local translation events that occur in response to external signals from dendritic spines. For example, mRNAs encoding β-actin, Arc, CaMKIIα, and BDNF are localized to dendrites and responsible for the structural and functional remodeling of dendritic spines upon neuronal activity [20]. Therefore, profiling the transcriptome in the synaptodendrosomes of AD mice can help capture the key mRNA involved in the regulation of dendrites and spines during neurodegeneration. To achieve this, we monitored the transcriptomic changes in synaptodendrosomes to elucidate the role of these synaptic transcripts in AD. In synaptodendrosomes, both the number of differentially expressed mRNAs and the magnitude of fold changes were greater in 6-month-old AD mice compared with 3-month-old AD mice. Functionally, these differentially expressed transcripts are involved in several key cellular processes, including mitochondrial metabolism, transmembrane transporter activity, ribosomal activity, and mRNA and protein binding. Furthermore, we identified the functions of three synapse-specific transcripts—Prnp, Cst3, and Cox6c—which are highly involved in the regulation of dendritic spine density and morphology. Overall, our study provides insights into the changes in synaptic mRNA and their significance during AD progression.

## 2. Results

### 2.1. Transcriptomic Profiling of Synaptodendrosomes in Alzheimer’s Disease Mice

**Figure 1:**
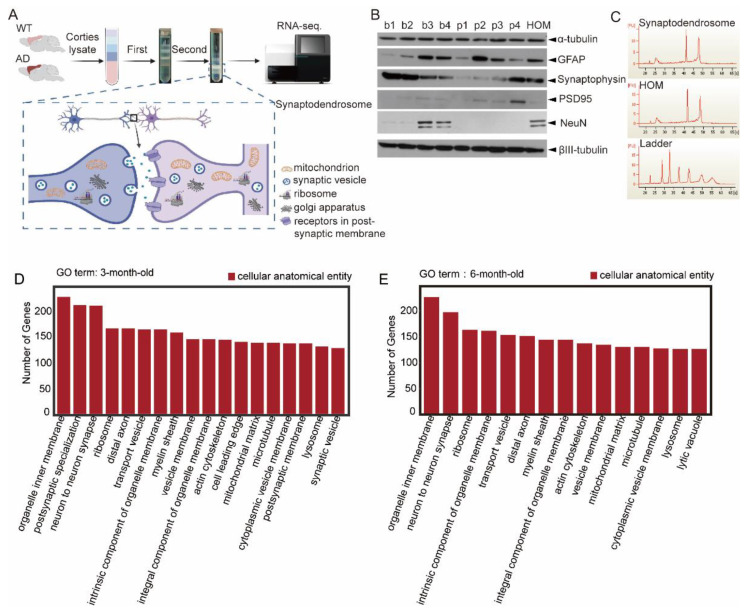
Synaptodendrosomes preparation and transcript identification. (**A**) Workflow of the synaptodendrosomes extraction procedure using 2-step density gradients. Cortical tissues were dissected from Alzheimer’s disease (AD) or wild-type (WT) mice at 3 or 6 months old, followed by subcellular fractionation to purify synaptodendrosomes. (**B**) Western blot analysis of all fractions generated in the preparation showed enrichment of synaptic markers (synaptophysin and PSD-95) in synaptodendrosomes fractions p3 and p4. b1–b4: first density gradient ultracentrifugation; p1–p4: second density gradient ultracentrifugation; HOM, homogenate. (**C**) Bioanalyzer analysis of RNA integrity in synaptodendrosomes (*n* ≥ 3 animals per group). (**D**) Gene Ontology (GO) terms whose enriched gene counts >130 were plotted and categorized as “cellular anatomical entity” or “protein-containing complex.” Samples were from 3-month-old WT mice. (**E**) GO terms whose enriched gene counts >130 were plotted and categorized as “cellular anatomical entity” or “protein-containing complex.” Samples were from 6-month-old WT mice. All transcripts with an average expression level of reads numbering ≥10 were used for GO enrichment analysis.

To investigate the localized changes in transcriptomic profiles implicated in synaptic dysfunction in AD, we prepared synaptodendrosomes from the cortices of APP/PS1 double-transgenic mice and wild-type (WT) littermates at 3 and 6 months old (Figure 1A). We confirmed the successful isolation of synaptodendrosomes fractions by examining the enrichment of the presynaptic marker synaptophysin and the postsynaptic marker PSD-95 in the p3 and p4 fractions while preparing the synaptodendrosomes of 3-month-old WT mice (Figure 1B). We measured the quality of RNA extracted from the synaptodendrosomes fractions before performing RNA sequencing (RNA-seq) (Figure 1C). Gene Ontology (GO) analysis of the transcripts identified in 3- and 6-month-old WT mice revealed that the most significant cellular components in synaptodendrosomes are closely associated with neuronal synapses, such as “organelle inner membrane”, “postsynaptic specialization”, “neuron to neuron synapse”, “ribosome”, “distal axon”, and “transport vesicle”, suggesting the successful enrichment of locally expressed genes in synaptodendrosomes (Figure 1D,E).

### 2.2. Changes in the Transcriptomic Profiles of Synaptodendrosomes in Alzheimer’s Disease Mice

**Figure 2:**
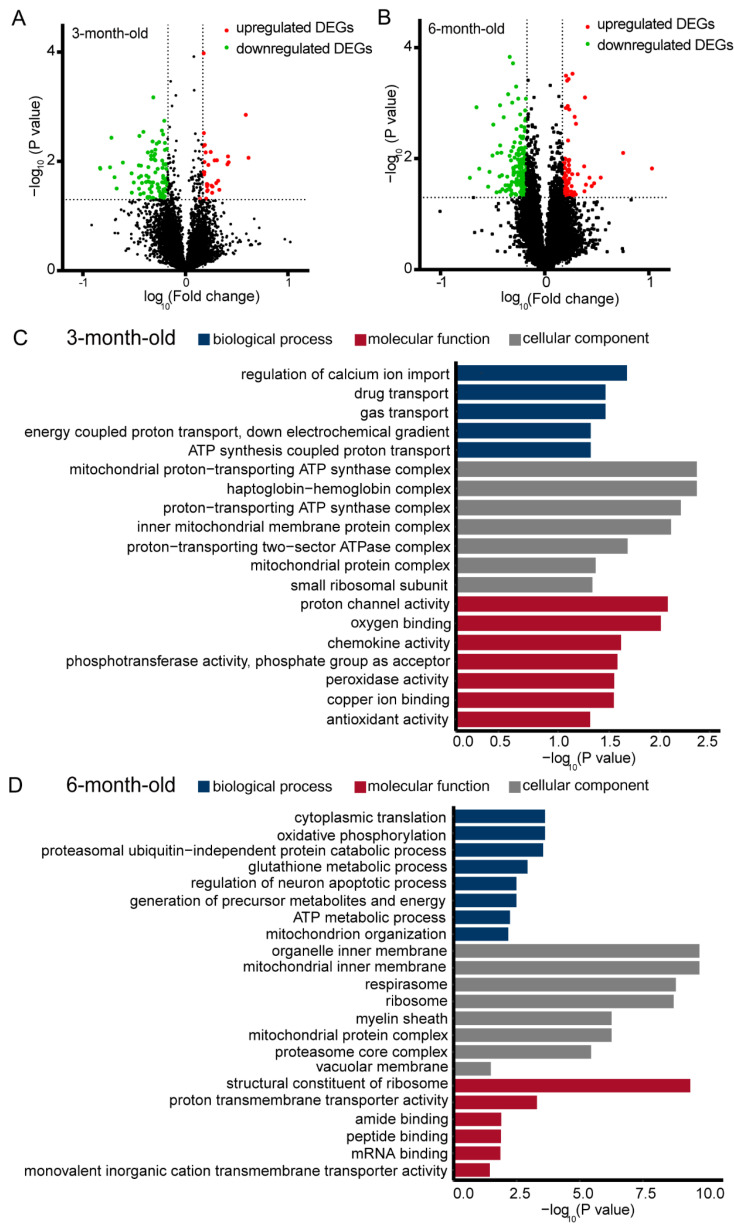
Differential gene expression in synaptodendrosomes of 3- and 6-month-old Alzheimer’s disease and wild-type mice. (**A**) Volcano plot of differentially expressed genes (DEGs) in 3-month-old mice (Alzheimer’s disease [AD] vs. wild-type [WT]; *p* < 0.05, fold-change ≥1.2 or ≤0.83). (**B**) Volcano plot of DEGs in 6-month-old mice (AD vs. WT; *p* < 0.05, fold-change ≥1.2 or ≤0.83). (**C**) The filtered DEGs from (**A**) were subjected to Gene Ontology (GO) enrichment analysis, and all 7 GO terms were selected (*p* < 0.05, fold-change ≥1.2 or ≤0.83). (**D**) The filtered DEGs from (**C**) were subjected to GO enrichment analysis, and the top 8 most-enriched functional GO terms of the DEGs were selected with “biological process (BP)”, “cellular component (CC)”, and “molecular function (MF)” *(p* ≤ 0.05, fold-change ≥1.2 or ≤0.83). All analyses were performed in a pooled sample from an average of *n* ≥ 3 animals.

We subsequently analyzed the changes in the transcriptomic profiles of synaptodendrosomes of AD mice. We found that 234 and 598 differentially expressed genes (DEGs) were significantly altered in the synaptodendrosomes of 3-month-old AD mice (*p* < 0.05, fold-change ≥1.2 or ≤0.83) (Figure 2A, Supplementary Table S1) and 6-month-old AD mice (Figure 2B, Supplementary Table S1), respectively. Functional cluster analysis of the DEGs in the synaptodendrosomes of 3-month-old AD mice identified the significant biological processes represented by the DEGs as “regulation of calcium ion import”, “drug transport”, “gas transport”, “energy coupled proton transport”, “down electrochemical gradient”, and “ATP synthesis coupled proton transport”, which are highly involved in the functions related to the establishment of the localization pathway, transport, and mitochondrial ATP synthesis (Figure 2C), demonstrating the functional specificity of the synaptically localized transcripts. GO analysis of the molecular function and cellular component further demonstrated that the DEGs were highly enriched in mitochondrial metabolism hemostasis, cation channels, and antioxidant activations (Figure 2C). GO analysis of 6-month-old AD mice showed that AD-related DEGs were enriched in energy metabolism activity, neuronal death, and mitochondrial organization—including cytoplasmic translation, oxidative phosphorylation, the proteasomal ubiquitin-independent protein catabolic process, the glutathione metabolic process, the ATP metabolic process, the generation of precursor metabolites and energy, mitochondrion organization, and the regulation of the neuron apoptotic process (Figure 2D). Our cellular component analysis results showed that these DEGs were associated with synaptically localized mitochondria, the organelle inner membrane, and the proteasome core complex (Figure 2D). In addition, these genes were involved in synaptic ribosome structure constitutions, iron transporter activity, and peptide and mRNA bindings within the biological process (Figure 2D). Furthermore, the numbers of DEGs were increased in 6-month-old AD mice compared with 3-month-old AD mice. Moreover, these discrepancy genes were mostly enriched in metabolic and neuronal apoptotic functions—such as oxidative phosphorylation, protein catabolism, glutathione metabolism, metabolites and energy, ATP metabolism, and neuron apoptosis in the biological process (Figure 2C,D). These results indicate that the dysregulation of energy metabolism may provide insights into AD pathogenesis.

### 2.3. Functional Diversity of Synaptodendrosomes-Localized mRNAs in Alzheimer’s Disease

**Figure 3:**
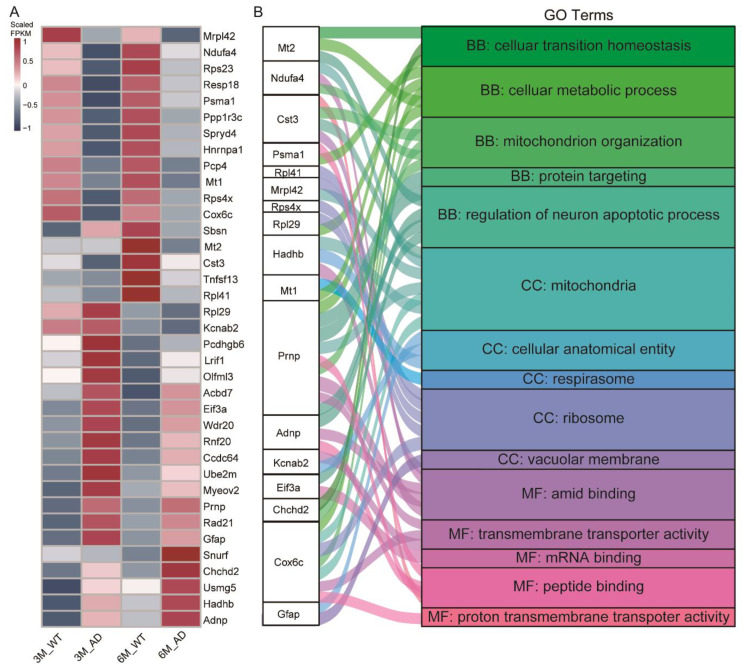
Local transcript changes associated with the mitochondrial metabolic signaling network. (**A**) Heatmap showing the differentially expressed genes (DEGs) that overlapped in both 3- and 6-month-old AD mice. Gene names of each group are listed. (**B**) Color spectrum showing the Gene Ontology (GO) terms associated with 17 DEGs of the top 10 enriched GO terms. The results are presented as a Sankey diagram, and the different colors indicate the relative GO terms respectively (*p* < 0.05, fold-change ≥1.25 or ≤0.8). The DEGs of samples from 6-month-old mice were ranked according to log2 (fold change), and the top 17 genes were selected. All analyses were performed in a pooled sample from an average of *n* ≥ 3 animals.

As we know, synaptic dysfunction occurs in early stage AD [21]. Changes in DEGs may provide mechanistic insights into the functional changes in synapses. Therefore, we identified 37 DEGs that overlapped the top 10 GO terms in both 3- and 6-month-old AD mice (Figure 3A). To functionally annotate the synaptically localized transcripts, we analyzed the overlapping DEGs and found that 17 of them are specifically associated with mitochondrial metabolic modulation among “molecular function”, “biological process”, and “cellular component” (Figure 3B). The GO analysis found that these 17 DEGs were expressed in mitochondria, respirasomes, ribosomes, and the vacuolar membrane, and were highly associated with the biological processes of “cytoplasmic translation” (Eif3a, Rpl29, and Rps23); “cellular metabolic process” (Rps23, Rpl29, Cox6c, Mt2, and Prnp); “mitochondria organization” (Cst3, Chchd2, Cox6c, Mt2, and Prnp); “regulation of neuron apoptotic process” (Prnp, Adnp, MT1, Ube2m, and Pcp4); and “protein targeting” (Prnp) (Figure 3B). Regarding their molecular functions, these genes were associated with the functions of binding site and transporter activity, including “amide binding” (Ndufa4, Cst3, Hadhb, Prnp, and Adnp); “transmembrane transporter activity” (Prnp, Kcna2, and Cox6c); “mRNA binding” (Eif3a); and “peptide binding” (Cst3, Prnp, and Adnp) (Figure 3B). Notably, the color spectrum of the GO analysis showed that Cst3, Prnp, and Cox6c predominantly localized in mitochondria and ribosomes (Figure 3B). These results suggest that most of the DEGs observed in the synaptodendrosomes of AD mice are enriched in mitochondrial metabolism, and our findings reveal the localized deregulation of synaptic transcripts during AD pathogenesis.

### 2.4. Alzheimer’s Disease-Associated Transcript Changes Reveal Dysregulated Synaptic Molecular Pathways in Synaptodendrosomes

**Figure 4:**
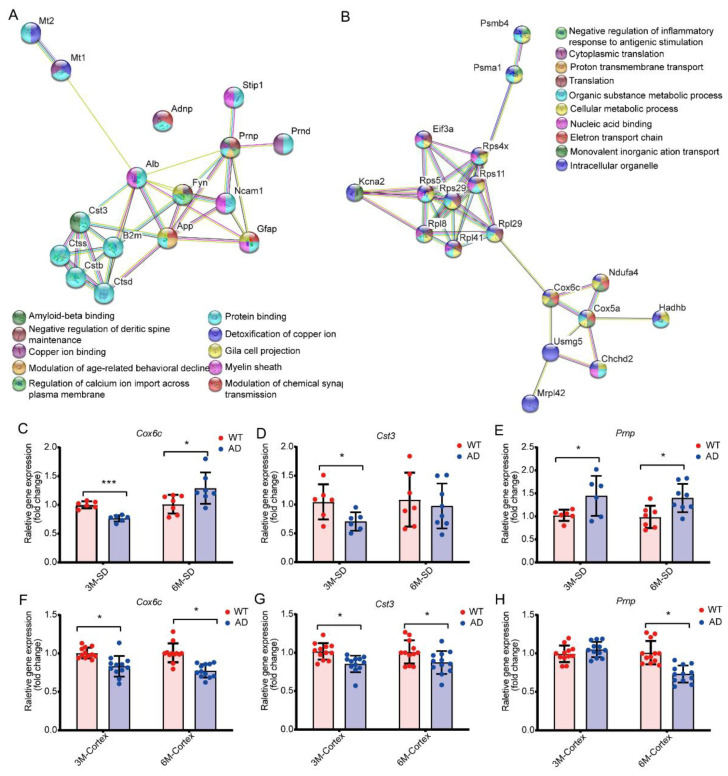
Protein–protein interaction network visualization and synaptically localized gene selection. (**A**,**B**) Identical genes from Figure 3B with significant changes (*p* < 0.05, fold-change ≥1.2 or ≤0.83) were used to generate a protein–protein interaction network by STRING v10. (**A**) Candidate genes were clustered in the “protein binding” and “chemical synaptic transmission” terms. (**B**) Candidate genes were enriched in metabolic and transport processes. (**C**–**H**) Regulation of synaptically localized differentially expressed genes was validated in either synaptodendrosomes or cortical tissues. All analyses were performed in 3 independent experiments (mean ± SEM; * *p* < 0.05; *** *p* < 0.001; two-way ANOVA; *n* ≥ 6 animals).

To better understand the biological roles of the 17 selected candidates, and how the alteration of these transcripts elicits their molecular mechanisms in AD, we conducted a protein–protein interaction (PPI) network analysis of the 17 AD-associated DEGs, which revealed two distinct clusters (Figure 4A,B). The PPI analysis showed that six DEGs (Mt2, Cst3, Gfap, Prnp, MT1, and Adnp) were enriched in one cluster and involved in 10 major functional pathways including amyloid binding, protein binding, negative regulation of dendritic spine maintenance, copper ion detoxification, copper ion binding, glial cell projection, modulation of age-related behavioral decline, and regulation of calcium ion import across the plasma membrane. Most of these DEGs were also closely associated with synaptic plasticity or AD pathogenesis (Figure 4A). The other cluster was enriched by the remaining 11 DEGs (Ndufa4, Rpl41, Mrpl42, Rps4x, Rpl29, Kcna2, Psma1, Hadhb, Eif3a, Chchd2, and Cox6c) which were involved in the following 10 major functional pathways: negative regulation of inflammatory response to antigenic stimulation, cytoplasmic translation, proton transmembrane transport, ribosomal translation, organic substance metabolic process, cellular metabolic process, nucleic acid binding, electron transport chain, monovalent inorganic action transport, and intercellular organelle. These DEGs were associated with the biological processes underlying the regulation of synaptic functions (Figure 4B).

We used quantitative real-time PCR (qRT-PCR) to investigate the expression of candidate genes. We investigated the expression of all 17 genes in the synaptodendrosomes of 3- and 6-month-old AD and WT mice (Supplementary Figure S1). To further understand the role of synaptodendrosomes-localized genes in the regulation of synaptic functions, we selected three candidate genes (*Cst3*, *Cox6c*, and *Prnp*) for further investigation, which were predominantly localized to mitochondria and ribosome regulation according to the GO and PPI analyses. The results showed that Cox6c was significantly downregulated in both the synaptodendrosomes and cortices in 3-month-old AD mice but exhibited the opposite pattern in cortical tissues with synaptodendrosomes in 6-month-old AD mice (Figure 4C,F). Cst3 was significantly downregulated in the cortical tissues of both 3- and 6-month-old AD mice (Figure 4G) but was only significantly downregulated in the synaptodendrosomes of 3-month-old AD mice (Figure 4D). Prnp was significantly upregulated in the synaptodendrosomes of both 3- and 6-month-old AD mice, compared with those of the age-matched WT controls (Figure 4H). These results suggest that the expression levels of *Cox6c, Cst3*, and *Prnp* in synaptodendrosomes and cortices may be differentially regulated during AD progression.

### 2.5. Reduced Expression of Cox6c, Cst3, or Prnp Alters Dendritic and Synaptic Morphology in Neurons

**Figure 5:**
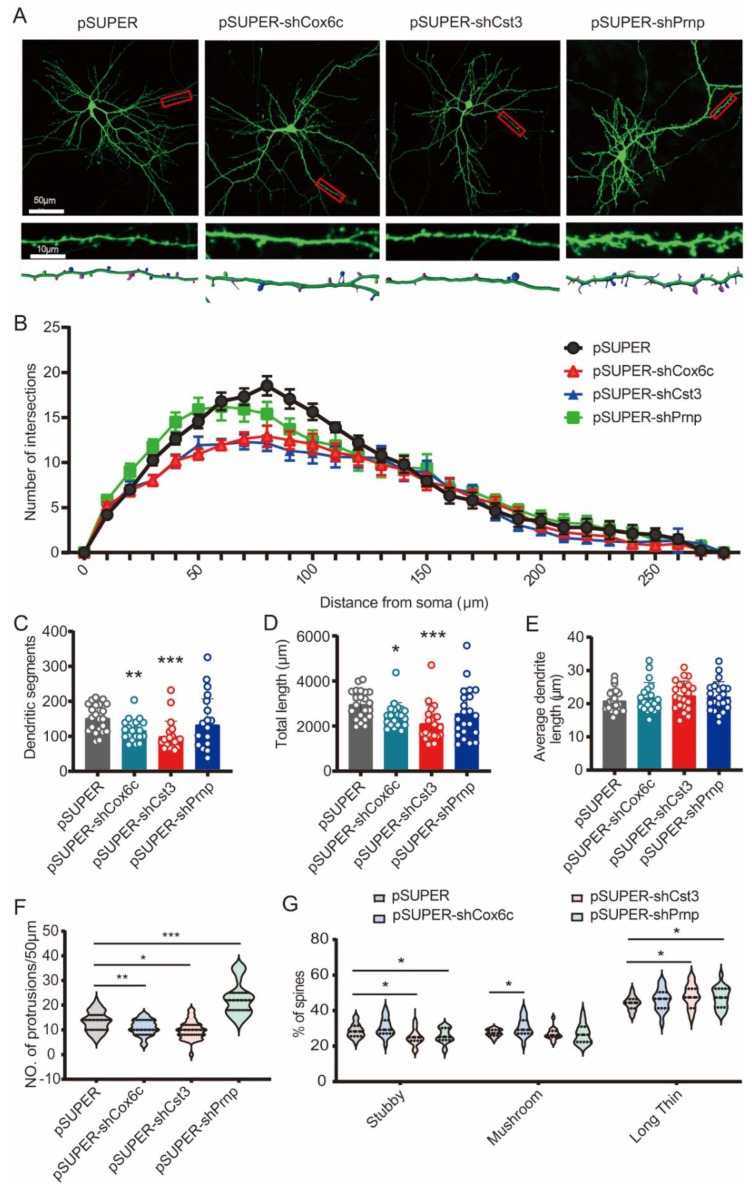
Downregulation of Cox6c, Cst3, or Prnp alters dendritic spine density and morphology. (**A**) Rat hippocampal neurons were transfected at 12 days in vitro (DIV) with pSUPER-GFP plus pSUPER-shCox6c, pSUPER-shCst3, and pSUPER-shPrnp, respectively. Confocal images (40×) were collected at DIV 14. Scale bar: (**top**), 50 μm; (**bottom**): 10 μm. Representative images of GFP in transfected neurons are shown, along with magnified images of the dendritic protrusions. Imaris 3D reconstructions used to quantitatively analyze dendritic spines are shown below. (**B**) Sholl analysis of dendritic arbors. (**C**) Numbers of dendritic segments quantification. (**D**) Analysis of total dendritic length. (**E**) Analysis of average dendritic length. (**F**) Quantitative analysis of dendritic spine densities (total of 20–25 cells from 3 independent experiments). (**G**) Spine classifications and quantification of stubby, mushroom, or long thin spine morphology using Imaris. Data presented in 3 independent experiments (from a total of 20–25 neurons). Data are mean ± SEM; * *p* ≤ 0.05, ** *p* ≤ 0.01, *** *p* ≤ 0.001; two-way ANOVA.

Disruption of the transport and local translation of dendritic mRNAs (including dendritic spine mRNAs) is involved in many neurodegenerative disorders, including AD [22,23]. To examine the roles of Cox6c, Cst3, and Prnp on dendritic architecture, we used two shRNAs against a specific gene and examined the knockdown efficiency in cultured hippocampal neurons (Supplementary Figure S2). We traced the dendritic arbors of the neurons by using shRNAs against individual transcripts, respectively transfected with GFP in Figure 5A (Figure 5A). Sholl analysis showed that the reduction in Cox6c and Cst3 inhibited dendritic arborization compared with control neurons (Figure 5B). This was accompanied by significant decreases in the numbers of dendritic segments (Figure 5C) and total dendritic length (Figure 5D) (* *p* ≤ 0.05, ** *p* ≤ 0.01, *** *p* ≤ 0.001). Interestingly, reduced Prnp expression in cultured neurons did not result in any significant differences in the dendritic arbors (Figure 5C,D). To further confirm the effects of Cox6c, Cst3, and Prnp downregulation on dendritic spine morphogenesis, we reconstructed dendritic arbors of neurons transfected as in Figure 5A. The analysis results showed that downregulation of either Cox6c or Cst3 in primary neurons led to a remarkably lower spine density (* *p* ≤ 0.05, ** *p* ≤ 0.01) (Figure 5F, Supplementary Figure S3A,B). Moreover, the reduction in Cox6c in cultured neurons significantly changed spine morphology and increased the percentage of mushroom dendritic spines (* *p* ≤ 0.05), while Cst3 knockdown significantly reduced the percentage of stubby spine morphology and increased long thin spine morphology (* *p* ≤ 0.05) (Figure 5F,G). Downregulation of Prnp in hippocampal neurons led to a higher spine density with decreased percentage of stubby spine morphology and increased the percentage of long thin spine morphology (* *p* ≤ 0.05, *** *p* ≤ 0.001) (Figure 5F,G, Supplementary Figure S3C). These data indicate that decreased Prnp expression identically affects dendritic spine density without changing the whole neuron morphology, while reduced Cox6c and Cst3 alter not only dendritic spine density but also the whole neuron phenotype in cultured hippocampal neurons. 

## 3. Discussion

Previous studies show that synaptodendrosomes contain membrane vesicles of synaptosomes, postsynaptic components, and substantial numbers of dendritic fragments [17,20]. Since synaptic failure is an important early sign of AD pathogenesis, synaptodendrosomes are used as an ex vivo model for studying synaptic disorder-related neurodegenerative diseases [24]. Synaptic functions and plasticity require mRNAs and their local translation in synapses [25]. Current evidence suggests that the individual synaptically localized mRNAs of CaMkIIa, β-actin, and PSD-95 are localized and translated at dendritic spines to maintain the diversity of synaptic plasticity [26]. However, the overall transcriptomic changes at synaptodendrosomes in the AD are largely unknown. In this study, we examined the local transcriptomic changes in synaptodendrosomes during AD pathogenesis. To the best of our knowledge, this is the first investigation of the local transcriptome in synaptodendrosomes in AD, which is documented as a leading research focus on synapse functions. 

Different mRNA transcripts display rapid and distinct dynamic properties at neuronal dendrites. mRNAs are localized and translated within dendrites and axons to provide mRNAs or proteins for remodeling and maintaining synapses [27]. Previous works demonstrate that many synaptically localized mRNAs are regulators of synaptic plasticity during AD pathogenesis. However, studies have not investigated the pool of local mRNA populations in AD synaptodendrosomes. To achieve this, we examined the transcriptomic profiles of the synaptodendrosomes at 3 and 6 months in WT controls and AD transgenic mice. 

A recent investigation of synaptosomes from 8-month-old AD mice showed a deterioration in mitochondrial functions, manifestation of apoptosis-related processes, and alterations in the organization of synaptic proteins, which are associated with complement-dependent synapse loss and further result in neuronal death in AD [28]. This is consistent with our findings in synaptodendrosomes, while our data further suggest that the deficits in mitochondrial functions can be observed in AD mice as early as 3 months old. Furthermore, the apoptotic-like processes appear in the synaptodendrosomes of AD mice as early as 6 months old. These results indicate that transcriptomic profiling in synaptodendrosomes may help capture some early signatures during disease progression. We also found that the DEGs were enriched in the mRNA binding and energy transport pathways in AD synaptodendrosomes. Deregulation of mRNA binding proteins and transporters in AD synaptodendrosomes can provide insights into the malfunctioning of long-distance transport of mRNA to dendrites, which allows local translation in response to external signals from dendritic spines. 

In addition, it was found that disruption of white matter integrity occurs in AD patients, implying that the genes involved in the myelination process are dysregulated during the progression of AD [29,30]. By comparing the gene expression of synaptodendrosomes from AD mice and its littermate control, we also observed the enrichment of DEGs regulating myelination, suggesting that aberrant myelination may also contribute to the deterioration of cognitive function in AD brain. This was further supported by a single cell transcriptomic analysis in prefrontal cortex of AD patients, which demonstrated that the expression of myelination-related genes was deregulated in many cell types, such as oligodendrocytes, neurons, and glia cells [1]. Particularly, neuronal expression of myelin-related gene PRNP gene was perturbed in AD brain, but how Prnp participates in AD pathogenesis remains unknown. In our study, we provided functional analysis by knockdown of Prnp expression in neurons, and further demonstrated that the expression of Prnp is required for synapse formation and maturation, supporting the notion that myelin-related genes regulate synaptic functions, deregulation of which may contribute to AD pathogenesis.

Consistent with previous reports, in synaptodendrosomes, mitochondria play critical roles in neuronal functions including synaptic neurotransmission and plasticity through mitochondrial swelling and energy metabolism (such as ATP generation) [31,32]. Mitochondrial and ribosomal dysfunction, the major transcriptional changes and early features of susceptible neurons, consequently cause oxidative stress, ATP production, and cytoplasmatic calcium concentration, leading to alterations in synaptic strength and plasticity in AD and Parkinson’s disease [1,33,34,35]. Indeed, our GO analysis results revealed that the accumulation of changes in mitochondria reduced mitochondrial membrane potential, leading to Ca^2+^ homeostasis disruption and oxidative stress induction within synaptodendrosomes [36]. APP/PS1 transgenic mice do not exhibit obvious cognitive impairment—in terms of either detectable brain Aβ plaques or perceptible synaptic loss—before 4 months old [37]. By comparison, AD phenotypes indicating obvious pathological progression—such as occasional Aβ deposits, synapse loss, and neuronal death—are observed after 6 months old [38,39,40,41,42,43]. Increased Aβ levels in neurons leads to the Aβ-dependent accumulation of mitochondrial Ca^2+^; this is observed prior to synapse loss and consequently results in neuronal apoptosis [44,45]. Consistent with the above evidence, our GO enrichment results indicate that Ca^2+^ dysregulation and ATP synthesis interruption occur in early stage AD (in 3-month-old AD mice). Furthermore, our transcriptomic results show that mitochondrial metabolism disorder and oxidative stress appear in early stage AD and gradually become more severe during AD progression [32,46,47]. These results indicate that the early pathological changes in synaptodendrosomes are linked to synaptic disorder in early stage AD [21]. 

Although synaptic dysfunction is considered as a pathological hallmark of AD [48], little is known about the function of local mRNAs in synaptodendrosomes modification of mitochondrial energy metabolism and synaptic structure and functions during AD pathogenesis. In this study, we discovered many synaptodendrosomes-localized transcripts that lead to synapse deficiency in early stage AD, including but not limited to Prnp, Cst3, and Cox6c [49,50,51]. In addition to identifying that mRNA expression dysregulation is involved in synaptic plasticity, our molecular characterization of synaptodendrosomes in AD transgenic mice investigated underlying gene knockdown in cultured primary neurons. Our gene expression data showed that synaptic dysregulation occurs with Prnp upregulation in the synaptodendrosomes of both 3- and 6-month-old AD mice. Synaptic compartments are enriched with protease-resistant proteins (PrPs) [52]. Accordingly, presynaptic and synaptophysin-positive PrPs colocalize with Aβ and tau in patients with AD [53]. A recent study comparing the transcriptomic profiles among AD-pathology, early-pathology, and no-pathology subgroups at the single-cell level in AD patients found that Prnp specifically regulates neuronal myelination during neurodegeneration [1]. Moreover, PrPc (cellular prion protein) binds Aβ oligomers at cell surfaces with high affinity, and multiple studies show that Prnp plays a central role in AD pathogenesis [54,55,56,57,58,59,60]. PrPc is involved in postsynaptic density localization by mediating the local effects of Aβ on synaptic plasticity, dendritic spine retraction, and synaptic loss [54]. Moreover, loss of PrPc results in decreased mitochondrial numbers and abnormal mitochondrial morphology, leading to changes in synapse structure and function [49]. Indeed, in our study, Prnp knockdown resulted in more dendritic spines, providing an explanation for the local administrative function of synaptic plasticity. As most patients with an AD-type pathology show PrPc–Aβ plaque colocalization, those overexpressing PrPc are more susceptible to Aβ cytotoxicity [61]. As such, Aβ/PrPc signaling pathway activation increases Aβ aggregation, induces cytotoxicity, and alters local dendritic spines in neurons, suggesting that Prnp reduction is a therapeutic target for early stage AD.

We found that the energy metabolism genes of Cst3 and Cox6c were expressed in the synaptodendrosomes of both AD and WT mice, but their expression levels differed between the two groups. Therefore, these findings raise the possibility that changes in the expression of Cst3 and Cox6c regulate local-translatability-mediated synaptic plasticity [62]. Cst3 is an endogenous cysteine protease inhibitor that is localized to the Golgi apparatus and lysosomes [63] and involved in programmed cell death [64]. Cst3 is the first cystatin found colocalized with accumulated Aβ in both amyloid-laden vascular walls and with parenchymal Aβ senile plaques [65,66]. Decreased secretion of cellular Cst3 provides a mechanism for increased risk of AD pathogenesis [67]. Another study identified that Cst3 removal directly inhibits cathepsin B-induced Aβ degradation associated with neuronal deficits in an AD mouse model [68]. Consistent with previous studies, we found that Cst3 levels were decreased in the synaptodendrosomes of 3-month-old AD mice. Moreover, we identified that knockdown of Cst3 in vitro decreases the number of dendritic spines, which may explain how Cst3 modulates synapse density in AD. In addition, the Cox6c protein is encoded in ribosomes after translation and is transported to mitochondria via different pathways [51]. Cox6c was found to be associated with neuronal damage and a modest reduction in protein levels in the brains of three patients with AD [69]. In contrast, increased mitochondrial function promoted increased Cox6c protein expression in a mouse model of ischemia [70]. Given that higher levels of Cox6c are necessary for the stability of mitochondrial electron transport membrane potential, a reduction in Cox6c may impair dendritic spine formation through the inhibition of mitochondrial activation. Furthermore, mitochondrial dysfunction contributes to dendritic spine loss by inducing changes in mitochondrial morphology and apoptosis. 

Taken together, our findings provide novel insights into the localized regulation of mRNAs of synaptodendrosomes in early stage AD. Our transcriptome analysis of synaptodendrosomes demonstrates a number of localized transcript aberrations in synaptodendrosomes in AD that may directly or indirectly lead to synaptic disorder. This study provides a molecular dissection of synaptodendrosomes-based therapeutic targets that could potentially restore neural homeostasis and ameliorate the pathological progression in early stage AD.

## 4. Materials and Methods

### 4.1. Animals

All experiments were approved by the Animal Care Committee of the Shenzhen Institute of Advanced Technology and conducted in accordance with the regulations. For transcriptomic profiling, we used male heterozygous, double-transgenic APP/PS1 mice from the Jackson Laboratory (B6C3-Tg [APPswe, PSEN1dE9] 85Dbo/J; stock number: 2010-0001) and WT littermates. We purchased SPF-class E14 pregnant Sprague Dawley (SD) rats from Beijing Vital River Laboratory Animal Technology Co., Ltd. (Beijing, China). We housed all animals in specific pathogen-free conditions with 50% humidity, a constant temperature of 23 °C, and a 12:12 h light/dark cycle.

### 4.2. Purification of Synaptodendrosomes

We homogenized and fractioned mouse cortices and subsequently purified synaptodendrosomes as previously described, with some optimizations [71,72,73]. Briefly, we dissected mouse brains under sterile conditions and rinsed the cortices in cold homogenization buffer containing: sucrose (320 mM), Tris-HCl (10 mM), EDTA (1 mM), DTT (2.5 mM), and NaF (0.25 mM). We homogenized the cortices in a glass tissue grinder of WHEATON^®^ Potter-Elvehjem Tissue Grinder (Cat. No. 358039; DWK Life Sciences, Mainz, Germany) using a homogenizer system (Cat. No. 099CK54; Glas-Col^®^, Terre Haute, IN, USA) containing 2 mL cold homogenization buffer. We performed homogenization with 18 gentle strokes in a tissue grinder with 0.1–0.15 mm clearance and a pestle driven at 1500 rpm for shearing action. We filtered the homogenate by a 30 µm nylon filter before mixing it with 50% OptiPrep solution (Cat. No. D1556-250; Sigma-Aldrich, St. Louis, MO, USA) to make a final concentration of 35% (10 mL). We then placed the solution in the bottom of a Beckman centrifugation tube, and layered 5 mL OptiPrep solutions (25%, 15%, 12.5%, and 9%) on top. Next, we centrifuged the solution for 24 min at 10,000× *g* in an ultracentrifuge (SW 41 Ti Rotor, LE-80K Ultracentrifuge; Beckman, Pasadena, CA, USA), and collected the synaptodendrosomes-containing fraction at the 9–12.5% interface. We diluted the above fraction 2 times in 1× phosphate-buffered saline (PBS) in a total volume of 10 mL and loaded on top a serial 5-mL sucrose–Percoll (Cat. No. P1644; Sigma-Aldrich) step gradient (6%, 10%, 15%, and 23%), followed by centrifugation for 9 min at 32,000× *g*. We then collected the synaptodendrosomes fraction from the 15–23% interface. Finally, we diluted the fraction in homogenization buffer and centrifuged it at 6000× *g* for 2 min.

### 4.3. RNA Extraction and High-Throughput Sequencing

We extracted the total RNA from both tissues and synaptodendrosomes fractions using TriZol (Cat. No. 15596026; Invitrogen Life Technologies, Carlsbad, CA, USA) according to the manufacturer’s instructions, followed by isopropanol precipitation. We stored the total RNA at −80 °C until use. 

For Illumina high-throughput sequencing, we prepared the cDNA library using the TruSeq Stranded mRNA Library Prep Kit (Cat. No. RS-122-2101; Illumina, San Diego, CA, USA). Briefly, mRNA was enriched using olig (dT) beads (Cat. No. 61005; Invitrogen Life Technologies) and fragmented randomly by the addition of fragmentation buffer. We then synthesized the cDNA using an mRNA template and random hexamer primer. Next, we added the second-strand synthesis buffer, dNTPs, RNase H, and DNA polymerase I to initiate second-strand synthesis. We then generated the double-stranded cDNA library through size selection and PCR enrichment. For quality control, we used Qubit 2.0 to test the library concentration, Agilent 2100 to measure insert size, and qPCR to quantify library efficiency. 

Next, we sequenced the cDNA library on an Illumina HiSeq 2000 platform with double-end reads by Novogene. We formatted the raw data as FASTQ files and aligned them against the GRCm38 reference genome using Bowtie2 with the default settings after pre-processing. Mapping reads were output as SAM files [74] and then tallied using htseq-count. 

### 4.4. Data Analysis

We filtered genes with expression level counts <10 prior to further analysis. We used the R package DESeq2 to perform differential expression analysis. We considered genes to be DEGs if they had a false discovery rate-adjusted *p*-Value <0.05 and log2 (fold change) >1.2 or <−0.833. We used GraphPad Prism 9 to explore the volcano plot and the R package Cluster Profiler to perform GO enrichment analysis of the DEGs, setting the *p*-Value cutoff at 0.05 [75]. We used the R package ggplot2 to visualize the results and STRING v10 to plot the PPI networks of the DEGs [76]. 

Quantitative data of the candidate gene expression, quantified neuronal dendrites, and dendritic spines were expressed as mean ± SEM and analyzed using GraphPad Prism 9. We determined the statistical significance of the differences between the 2 groups by a one-sample *t*-test or two-way ANOVA where appropriate and set the level of statistical significance at *p* < 0.05 (2-tailed).

### 4.5. Quantitative Real-Time PCR

We investigated the expression of selected genes by quantitative real-time PCR (qRT-PCR). We extracted the total RNA from both tissues and synaptodendrosomes fractions using TriZol, and synthesized cDNA using the RevertAid H Minus First Strand cDNA Synthesis Kit (Cat. No. K1632; Qiagen, Hilden, Germany) from extracted RNA according to the manufacturer’s protocol. Then, we performed qRT-PCR using a Bio-Rad CFX96 Touch™ Real-Time PCR Detection System (Cat. No. CFX96; Bio-Rad, Hercules, CA, USA). We designed gene-specific primers (Table 1 and Supplementary Table S2) online using Primer3 (version 0.4.0). At the end of the PCR reaction, we performed a melting curve analysis to detect the specificity of the PCR reaction. We measured each sample in 2 independent experiments with triplicates in each experiment. Relative mRNA expression was normalized to the housekeeping gene glyceraldehyd-3-phosphat-dehydrogenase (*GAPDH*). We analyzed gene expression by the comparative 2^−ΔΔCT^ method. 

### 4.6. Western Blotting

We examined the protein expression in synaptodendrosomes and other fractions from homogenate samples by Western blotting. Each band of homogenates was punched and lysed on ice in RIPA buffer (50 mM Tris [pH 7.6], 150 mM NaCl, 1% NP-40, 0.5% sodium deoxycholate, and 0.1% SDS) with a protease inhibitor cocktail (Cat. No. 11873580001; Roche, Basel, Switzerland). We centrifuged the lysates at 12,000 rpm at 4 °C for 10 min and quantified the proteins in the supernatants using a Pierce BCA Protein Assay Kit (Cat. No. 23225; Thermo Scientific, Waltham, MA, USA). We subjected equal amounts of proteins to SDS-PAGE and probed the membranes with specific antibodies including primary anti-tubulin (Cat. No. Ab195352; Abcam, Cambridge, UK), anti-GFAP (Cat. No. Ab174124; Abcam), anti-synaptophysin (Cat. No. Ab103328; Abcam), anti-PSD95 (Cat. No. Ab122340; Abcam), anti-NeuN (Cat. No. Ab104224; Abcam), and anti-βIII-tubulin (Cat. No. T8578; Sigma-Aldrich) at 1:1000 dilution overnight at 4 °C. We applied secondary antibodies conjugated with horseradish peroxidase to detect reactive bands; these antibodies were developed with the Amersham ECL Plus Detection Kit (GE Health, Chicago, IL, USA). 

### 4.7. Short Hairpin RNA Construction

We designed two short hairpin RNAs (shRNAs) against Cox6c, Cst3, and Prnp; these shRNAs were synthesized by Life Technologies (Table 2) and then cloned into the pSUPER vector. 

### 4.8. Primary Neuron Culture and Cell Transfection

We isolated and cultured hippocampal neurons from embryonic day 18–19 (E18–E19) rat embryos as previously reported [77,78]. Briefly, we dissected the E18–E19 rat hippocampi and collected them in HBSS buffer (Cat. No. 1470-112; Life Technologies) supplied with 10mM HEPES (Cat. No. 15630-080; Life Technologies), 1mM sodium pyruvate (Cat. No. 11360-081; Life Technologies), 0.09% D-glucose (Cat. No. G8769; Sigma-Aldrich), 0.09% D(+)-Glucose(+) (Cat. No. G7021; Sigma-Aldrich), 2mM L-glutamine (Cat. No. 25030-081; Life Technologies), and 1% Pen-Strep (Cat. No. A5955; Sigma-Aldrich). The tissues were minced, rinsed twice with F10 medium, and dissociated with 0.25% trypsin-EDTA (Cat. No. 25300-054; Life Technologies) for 20 min at 37 °C. We inactivated trypsin by trypsin inhibitor (Cat. No. T6522; Sigma-Aldrich). We obtained dissociated cells and centrifuged them at low speed (500 rpm) for 30 sec. We plated neurons on coverslips coated with poly-D-lysine (50 μg/mL) (Cat. No. P0899; Sigma-Aldrich) at 10,000 cells/mm^2^. We then cultured the neurons in neurobasal medium with 2% B27 and 0.5 mM L-glutamine supplementation. At 12 DIV, we transfected the cultured primary neurons with candidate plasmids for 48 h using calcium phosphate precipitation for an immunocytochemical assay [77]. 

### 4.9. Confocal Imaging and Morphological Analysis 

To image the morphological changes in dendritic spines in shRNA-transfected hippocampal neurons, we fixed neurons after 2 days of transfection with either 4% paraformaldehyde or 5% sucrose at room temperature as previously described [78]. We obtained fluorescence images under a Zeiss LSM900 scanning confocal microscope with 40× oil immersion objective. The neuronal body and dendritic spines were represented by Imaris 9.6 (Oxford, UK). We performed Sholl analysis and counted the number of spines and dendritic length on three-dimensional (3D) stacks using the Imaris measurement tool. All data are presented as mean ± SEM. We performed statistical comparisons using two-way ANOVA analysis in GraphPad Prism 9. 

## 5. Conclusions

This study provides a comprehensive analysis of the transcriptomic profiles of synaptodendrosomes in an AD mouse model. We found several transcript changes associated with mitochondrial metabolism in synaptodendrosomes and determined that these changes may contribute to synaptic dysfunction. Furthermore, the identification of synaptodendrosomes-engaged transcripts offers synaptically localized targets for the therapeutic intervention of neuropathological diseases. 

## Figures and Tables

**Table 1:** Primers used for real-time quantitative polymerase chain reaction analysis.

Target Gene	Directions	Sequences	Accession No.	Product Size
*Cox6c*	Forward	gggaaggacgttggtgtaga	NM_053071.2	111
	Reverse	ccagcaatatgaacccgcag		
*Cst3*	Forward	atacaggtggtgagagctcg	NM_009976.4	147
	Reverse	tgccttcctcatcagatggg		
*Prnp*	Forward	accagaacaacttcgtgcac	NM_001278256.1	177
	Reverse	ttctcccgtcgtaataggcc		
*Gapdh*	Forward	tcaacagcaactcccactcttcca	NM_001289726.1	115
	Reverse	accctgttgctgtagccgtattca		

**Table 2:** Short hairpin RNA primers.

Target Gene	Sequence (5′->3′)
ShCox6c #1	GCAGATTTCTACAGGAATT
ShCox6c #2	GGAATTATGACTCCATGAA
ShCst3 #1	GCAGCTTGTGGCTGGAATA
ShCst3 #2	GCCGAACTACATGTACCAA
ShPrnp #1	CCTGTGATCCTCCTCATCT
ShPrnp #2	TCCTCATCTCCTTCCTCAT

## Data Availability

The RNA-seq data reported in this article have been deposited in the Gene Expression Omnibus (GEO) at the National Center for Biotechnology Information (NCBI) and are accessible through the GEO Series accession number: PRJNA851549. All study data are included in the article and supporting information.

## Supplementary Materials

The following supporting information can be downloaded at: https://www.mdpi.com/article/10.3390/ijms23168888/s1.

## Abbreviations

AD	Alzheimer’s disease
DEGs	differentially expressed genes
GO	Gene Ontology
Aβ	amyloid beta
p-tau	hyperphosphorylated tau
NFTs	neurofibrillary tangles
RNA-seq	RNA sequencing
WT	wild-type
PrPs	protease-resistant proteins
HOM	homogenate
PPI	protein–protein interaction
SD	Sprague Dawley
qRT-PCR	quantitative real-time PCR
DIV	days in vitro
3D	three-dimensional
GAPDH	glyceraldehyd-3-phosphat-dehydrogenase
