# Supplementary material for: Early Changes in Transcriptomic Profiles in Synaptodendrosomes Reveal Aberrant Synaptic Functions in Alzheimer’s Disease
Source: Int J Mol Sci. 2022 Aug 10;23(16):8888. doi: 10.3390/ijms23168888 (PMC9408306; doi:10.3390/ijms23168888)
Supplement: Supplementary file 1 [file ijms-23-08888-s001.zip › ijms-1807443-supplementary.pdf]

Early Changes in Transcriptomic Profiles in Synaptodendrosomes Reveal Aberrant Synaptic Functions in Alzheimer's Disease

Xueqi Qu [1], Li Lin [1], Wanying Yi [2], Changyu Sun [2], Yuewen Chen [1,2,3] and Yu Chen [1,2,3,*]

[1] Chinese Academy of Sciences Key Laboratory of Brain Connectome and Manipulation, Shenzhen Key Laboratory of Translational Research for Brain Diseases, The Brain Cognition and Brain Disease Institute, Shenzhen Institute of Advanced Technology, Chinese Academy of Sciences, Shenzhen–Hong Kong Institute of Brain Science–Shenzhen Fundamental Research Institutions, Shenzhen 518055, China

[2] Guangdong Provincial Key Laboratory of Brain Science, Disease and Drug Development, HKUST Shenzhen Research Institute, Shenzhen 518057, China

[3] University of Chinese Academy of Sciences, Beijing 100049, China

[*] Correspondence: yu.chen@siat.ac.cn; Tel.: +86-755-2692-5498

This document includes:

Supplementary Figure S1 to S3 and Supplementary table S1 and S2

Supplementary Figure S1

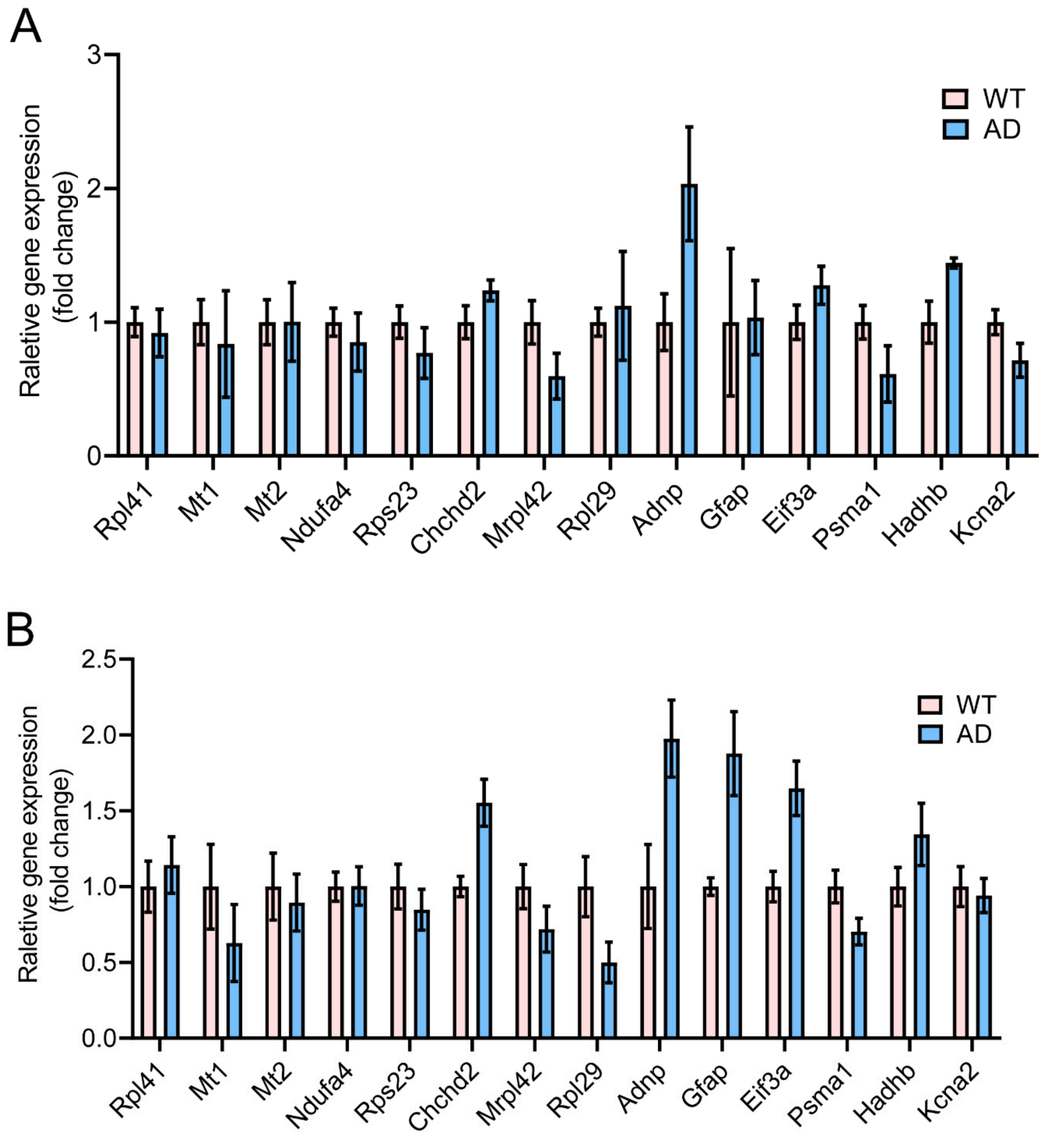

Supplementary Figure S1: Validation of candidate gene expression. We used quantitative RT-PCR to investigate the expression of candidate genes. (**A**) Differentially expressed genes were validated in synaptodendrosomes from 3-month old AD and WT mice. (**B**) Differentially expressed genes were validated in synaptodendrosomes from 6-month old AD and WT mice. All analyses were performed in 3 independent experiments (mean ± SEM; *$p < 0.05$, **$p < 0.01$, ***$p < 0.001$; two-way ANOVA; $n \geqslant 6$ animals).

Supplementary Figure S2

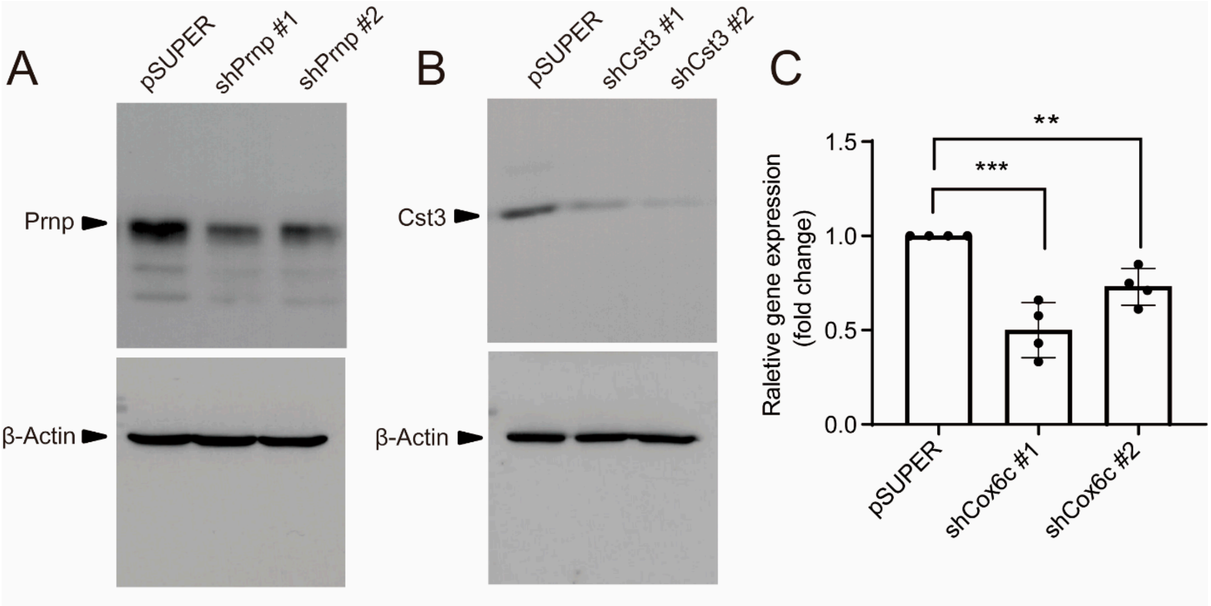

Supplementary Figure S2: Validation of knockdown efficiency of shRNAs. We designed 2 shRNAs against a specific gene. The knockdown efficiency was examined in cultured hippocampal neuron. (**A**) Western blot analysis of shPrnp #1 and shPrnp #2 efficiency. (**B**) Western blot analysis of shCst3 #1 and shCst3 #2 efficiency. (**C**) Due to the lack of commercially available Cox6c antibody to detect its expression in neurons, qPCR was used to validate the reduction of *Cox6c* transcript upon shRNA-mediated knockdown. Western blotting was performed in 2 independent experiments. qPCR was performed in 2 independent experiments with duplicates (mean ± SEM; $**p < 0.01$, $***p < 0.001$; two-way ANOVA; $n \geqslant 4$).

Supplementary Figure S3

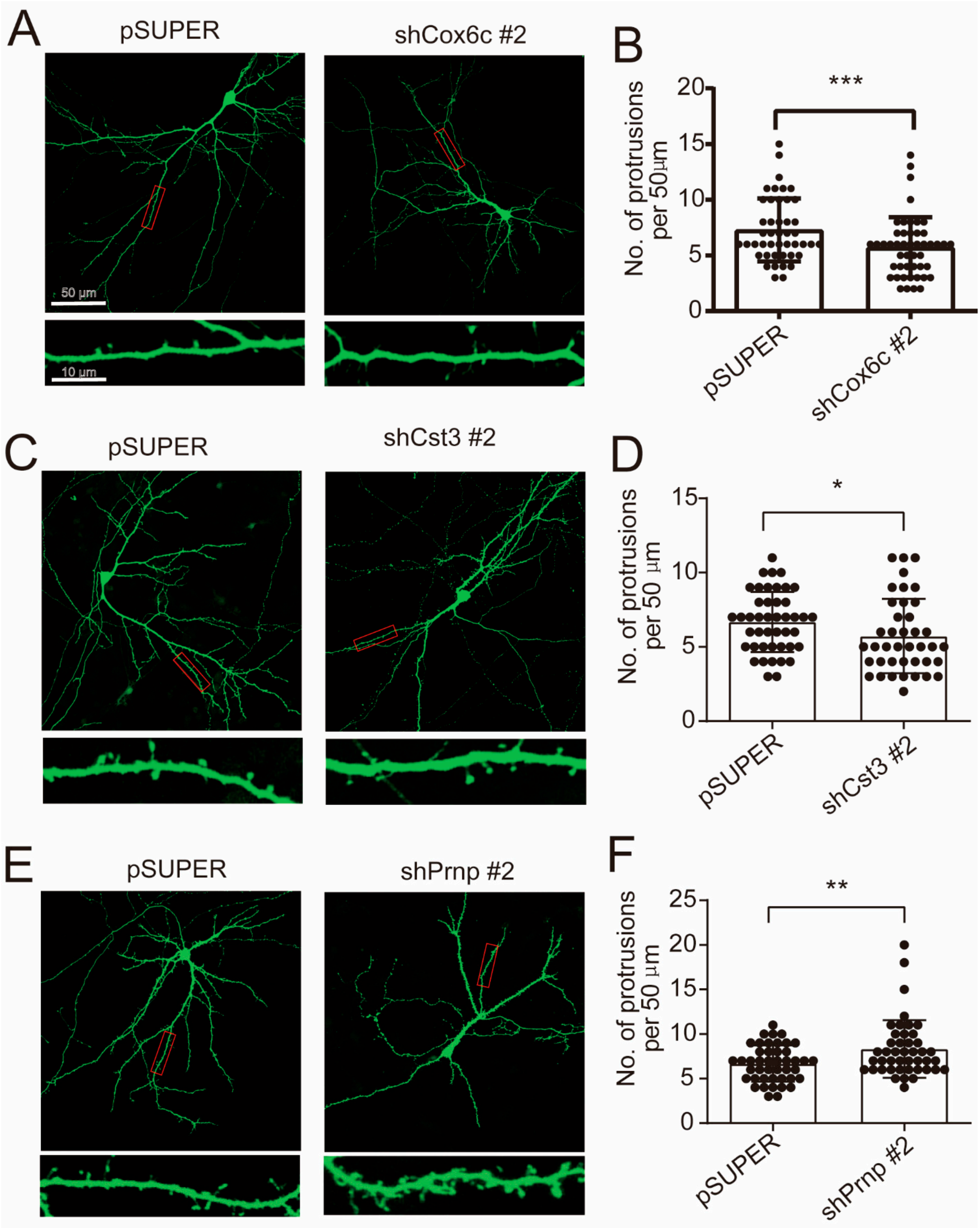

Supplementary Figure S3: Examination of neuronal morphology by shRNA-mediated knockdown of DEGs. We designed 2 shRNAs against a specific gene. The effect of the second shRNAs were shown here. (**A, C, E**) Rat hippocampal neurons were transfected at 12 days in vitro (DIV) with pSUPER-GFP plus pSUPER-shCox6c, pSUPER-shCst3, and pSUPER-shPrnp, respectively. Confocal images (40x) were collected at DIV 14. Scale bar: top, 50 µm; bottom, 10 µm. Representative images of GFP in transfected neurons are shown, along with magnified images of

the dendritic protrusions. (**B, D, E**) Quantitative analysis of dendritic spine density in Cox6c, Cst3 and Prnp-knockdown neurons, respectively. A total of 20–25 cells from 3 independent experiments (mean ± SEM; *$p < 0.05$, **$p < 0.01$, ***$p < 0.001$; two-tailed Student-t test).
