# Supplementary material for: Early Changes in Transcriptomic Profiles in Synaptodendrosomes Reveal Aberrant Synaptic Functions in Alzheimer’s Disease
Source: Int J Mol Sci. 2022 Aug 10;23(16):8888. doi: 10.3390/ijms23168888 (PMC9408306; doi:10.3390/ijms23168888)
Supplement: Supplementary file 1 [file ijms-23-08888-s001.zip › Qu et al._Supplementary Table S2_Primers list.pdf]

Supplementary Table S2. Primers for real-time quantitative polymerase chain reaction analysis.

Target Gene	Directions	Sequences	Accession No.	Product Size
Rpl41	Forward	ggaagaagagaatgcgcagg	NM_018860.4	142
	Reverse	gcacacagtccgatagcttg		
Usmg5	Forward	agaaaactccagctgcgaaa	AJ290947.1	216
	Reverse	atggggattggtatggagcc		
Mt1	Forward	ggacaggaagcagaggtctt	NM_013602.3	155
	Reverse	gttcgtcacatcaggcacag		
Mt2	Forward	cttttgcgctcgacccaata	NM_008630.2	224
	Reverse	cagcagcttttcttgcagga		
Ndufa4	Forward	gcatcccagcttgattcctc	NM_010886.3	141
	Reverse	cccagtttgttccatggctc		
Rps23	Forward	aaagtaggggttgaggccaa	NM_024175.3	205
	Reverse	ccttaaagcggactccagga		
Chchd2	Forward	aagcccgacatcacttacca	NM_024166.6	132
	Reverse	ctcacagagcttgacatcgc		
Mrpl42	Forward	tcccctacgaacacaccaaa	NM_026065.4	135
	Reverse	gttctatcatgggcccttgc		
Rpl29	Forward	gcacaacaagaaaggcctga	NM_001324534.1	120
	Reverse	ccttttggcatcttgggctt		
Adnp	Forward	gcgttcacttcgaaaaggag	NM_001310086.1	171
	Reverse	aacggcaatacggacaagac		
Gfap	Forward	agaaaaccgcatcaccattc	NM_010277.3	184
	Reverse	tcacatcaccacgtccttgt		
Eif3a	Forward	actccctgggtcaagttcct	NM_010123.3	157
	Reverse	cacatagcttgcggaactca		
Hnrnpa1	Forward	aaccgacgagagtctgagga	NM_010447.5	157
	Reverse	gtgtggtcttgcattcatgg		
Psma1	Forward	tggagtgcaatttggatgaa	NM_011965.2	245
	Reverse	cattggttcatcggcttttt		
Hadhb	Forward	gagctgttcttcccaactgc	NM_001289798.1	182
	Reverse	aacccgaaagtgcagctcta		
Tnfsf13	Forward	tcaggtggtatctcgggaag	NM_023517.2	195
	Reverse	aatgttccatgcggagaaag		
Kcna2	Forward	cccaagaaacggatgaggta	NM_008417.5	206
	Reverse	tcatcctcccgaaacatctc		
Ppp1r3C	Forward	gtggggactgacaccagagt	NM_016854.2	231
	Reverse	agtacatgcacaccggacaa		
Pcp4	Forward	ggtgaatgcctctcattggt	NM_008791.3	151
	Reverse	actcatgttggcctgactcc		
Ube2m	Forward	ctcagcgatgggaaatgaat	NM_145578.3	185

	Reverse	aggccatgcagtaacattcc		
Rnf20	Forward	cgacattgtgagctggagaa	NM_001163263.1	178
	Reverse	gggctttcaactgcagact		
Rif1	Forward	cctgccaattttgaacaggt	NM_175238.6	232
	Reverse	tcatttccagtcccaaaagc		
